# The modulation of acetic acid pathway genes in *Arabidopsis* improves survival under drought stress

**DOI:** 10.1038/s41598-018-26103-2

**Published:** 2018-05-18

**Authors:** Sultana Rasheed, Khurram Bashir, Jong-Myong Kim, Marina Ando, Maho Tanaka, Motoaki Seki

**Affiliations:** 1 Plant Genomic Network Research Team, RIKEN Center for Sustainable Resource Sciences, 1-7-22 Suehiro-cho, Tsurumi-ku, Yokohama, Kanagawa 230-0045 Japan; 2 Kihara Institute for Biological Research, Yokohama City University, Yokohama, 244-0813 Japan; 3 CREST, JST, 4-1-8 Honcho, Kawaguchi, Saitama 332-0012 Japan; 4 Plant Epigenome Regulation Laboratory, RIKEN Cluster for Pioneering Research, 2-1 Hirosawa, Wako, Saitama 351-0198 Japan

## Abstract

The *Arabidopsis histone deacetylase 6* (*HDA6*) mutant exhibits increased tolerance to drought stress by negatively regulating the expression of *ALDH2B7* and *PDC1*. Therefore, it was logical to determine if transgenic *Arabidopsis* plants expressing *PDC1* or *ALDH2B7* using a suitable promoter would also exhibit tolerance to drought stress. An analysis of published microarray data indicated the up-regulation of the *TSPO* gene, which encodes an outer membrane tryptophan-rich sensory protein (TSPO), by drought stress. RT-qPCR, as well as GUS analysis of the promoter, confirmed the up-regulation of *TSPO* by drought stress in *Arabidopsis* roots and shoots. Thus, the *TSPO* promoter was used to drive drought-responsive expression of *ALDH2B7* and *PDC1*. RT-qPCR analysis confirmed that the expression of *PDC1* and *ALDH2B7* was up-regulated, relative to WT plants, by drought stress in homozygous *pTSPO-PDC1* and *pTSPO-ALDH2B7* plant lines. *pTSPO-ALDH2B7* and *pTSPO-PDC1* transgenic lines showed prolonged survival under drought stress. Microarray analyses revealed transcriptomic changes related to metabolism in *pTSPO-PDC1* plants, indicating that selective regulation of metabolism may occur; resulting in the acquisition of drought stress tolerance. These results confirmed that *TSPO* promoter can be used to elevate the expression of acetic acid biosynthesis pathway genes; ensuring prolonged survival under drought stress in *Arabidopsis*.

## Introduction

Drought stress negatively impacts photosynthesis and plant growth and can ultimately result in reduced crop yield and enormous economic losses. As a result, the development of drought tolerant crops has received great attention and aims to mitigate the deleterious effects associated with drought stress on crop production. The molecular response to drought stress has been comprehensively characterized^1,2^, including the transcriptomic changes that occur in roots and shoots of soil grown, drought-stressed *Arabidopsis* plants^3,4^. These various analyses led to the identification of genes that are putatively involved in plant response to drought stress. Therefore, these data could be potentially utilized to genetically engineer drought tolerant plants by transforming plants with novel combinations of genes and promoters. In this regard, *Arabidopsis histone deacetylase 6 (HDA6)* mutants are tolerant to drought stress^5,6^. HDA6 negatively regulates the expression of *PDC1* & *ALDH2B7*, as indicated by the up-regulated expression of *PDC1* and *ALDH2B7* in the *hda6* mutant^5^.

**Figure 1:**
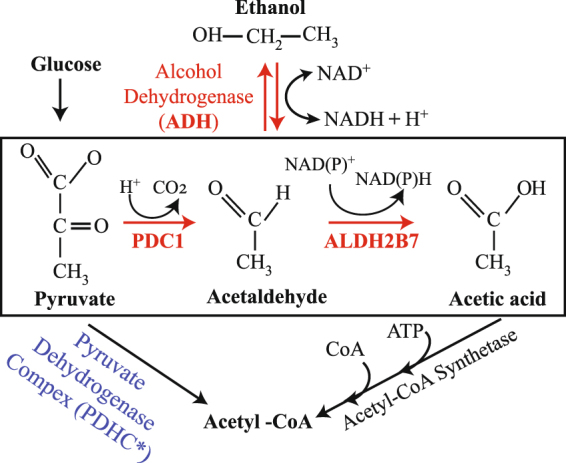
The acetic acid biosynthesis pathway in plants. Pyruvate is converted to acetaldehyde by PDC1 and the resultant acetaldehyde is converted to acetic acid by ALDH2B7. Acetaldehyde may also be converted to ethanol and *vice versa*. According to *Rasheed et al*. (2016a), genes shown in red were up-regulated in response to drought stress, while the genes shown in blue were down-regulated. *The expression of only the mitochondrial PDH-E1 alpha (*AT1G01090*) and beta (*AT1G30120*) subunits decreased significantly in response to drought stress.

In *Arabidopsis*, *PDC1* and *ALDH2B7* genes have functional roles in the acetic acid fermentation pathway (Fig. 1). Pyruvate dehydrogenase (PDH) converts pyruvate into the Acetyl-Co enzyme A (Acetyl-CoA) and is termed the PDH pathway. Acetyl-CoA can also be synthesized by an alternative pathway in which PDH is bypassed. In this case, PDC proteins convert pyruvate to acetaldehyde, which is subsequently converted to acetate by ALDH enzymes^7,8^. The PDC family in *Arabidopsis* is comprised of 4 members (PDC1, PDC2, PDC3 & PDC4). PDC proteins convert pyruvate to acetaldehyde, and thus contribute to ethanol fermentation, as well as acetic acid production. PDC1 is 607 amino acids in length and is predicted to localize to the cytoplasm; although its localization has not been verified. Among the other PDC proteins in *Arabidopsis*, PDC1 shares 89% homology with PDC4, 88% with PDC3, and 82% with PDC2. *Arabidopsis* PDC proteins have been reported to play a role in ethanol fermentation^9,10^. Their role in acetate production through a PDH independent pathway, however, has never been verified or comprehensively discussed.

The aldehyde dehydrogenase (ALDH) superfamily is a diverse family of proteins controlling aldehyde metabolism. ALDH enzymes convert aldehydes to their corresponding carboxylic acids utilizing NAD^+^ or NADP^+^ as a cofactor to yield NADH or NADPH. In contrast to PDC family proteins, which are confined to plants and fungi, the ALDH family is ubiquitous in prokaryotic and eukaryotic organisms; including plants^11^. Aldehyde compounds play an essential role in several catabolic and biosynthetic pathways and also contribute to the maintenance of the cellular redox balance^8,11–13^. Plant ALDHs perform a diverse array of functions in many catabolic and biosynthetic pathways, including amino acid metabolism, glycolysis, and carnitine biosynthesis^8,11,12^. In plants, several studies have reported the up-regulation of *ALDH* genes in response to a variety of stress conditions. Overexpression of *ALDH* genes can increase stress tolerance^14,15^. In general, the expression of *ALDH* genes in *Arabidopsis* is not regulated by anoxia. Rather, the expression of *ALDH2B7* is up-regulated in response to ABA application and dehydration; suggesting that ALDH may play a role in aerobic detoxification of acetaldehyde. Additionally, in response to stress, ALDH activity may occur independent of ethanol fermentation^9^.

Microarray analyses have indicated that the expression of *PDC1* and *ALDH2B7* are up-regulated by drought stress. These data, combined with the drought tolerant phenotype exhibited by the *hda6* mutant and the enhanced drought tolerance that is induced by the exogenous application of acetic acid^5^, strongly suggest that plants expressing *PDC1* or *ALDH2B7* under the control of a suitable promoter could significantly enhance drought stress tolerance.

In order to be of practical utility, drought tolerant plants should not exhibit any undesirable phenotypes^16,17^. Avoiding undesirable, non-target, responses could be achieved by regulating the expression of genes that convey drought tolerance through the selection of a promoter that regulates the desired genes in a strict spatial and temporal manner. In this case, an ideal promoter should induce gene expression in roots and shoot organs specifically in response to drought stress, particularly during the early phase of drought stress. An analysis of recent microarray data indicated that the expression of a gene encoding a tryptophan-rich sensory protein (*TSPO*) is significantly up-regulated in roots and shoots during early drought stress^3^. TSPOs are small ubiquitous transmembrane proteins that are found in prokaryotes, plants, animals, and humans^18^. *Arabidopsis* TSPO localizes to the Golgi apparatus and the expression of the *TSPO* gene, as well as the accumulation of the protein, is strictly regulated by abiotic stresses and ABA^19–21^. These data indicate that the *TSPO* promoter could potentially be utilized for regulating genes in transgenic plants that convey drought tolerance. Thus, transgenic *Arabidopsis* plants expressing *PDC1* or *ALDH2B7* genes under the control of the *TSPO* promoter (*pTSPO*-*PDC1* or *pTSPO*-*ALDH2B7*) were developed. The resulting transgenic plants exhibited a significant increase in drought stress tolerance. These results demonstrate that the elevated expression of acetic acid biosynthesis pathway genes using a *TSPO* promoter can significantly enhance drought stress tolerance in *Arabidopsis* plants.

## Results

### Analysis of drought-inducible expression of *PDC1*, *ALDH2B7*, and *TSPO* genes

**Figure 2:**
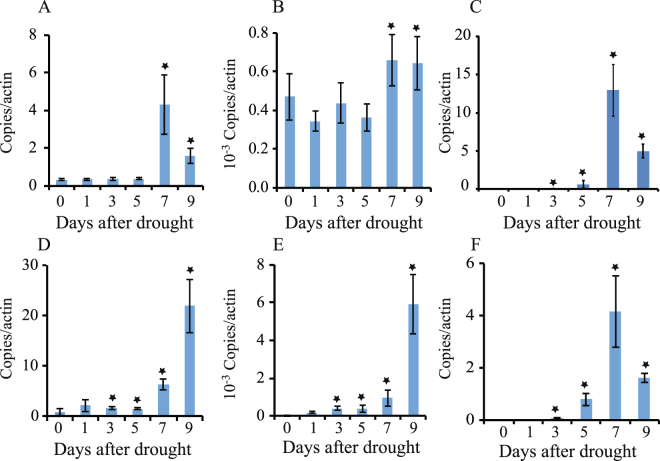
RT-qPCR confirmation of up-regulation of *ALDH2B7*, *PDC1*, and *TSPO* in response to drought stress in WT plants. (**A**) Expression of *PDC1*, (**B**) *ALDH2B7*, and (**C**) *TSPO* in shoots of *Arabidopsis* plants grown in ceramics soil. (**D** Expression of *PDC1*, (**E**) *ALDH2B7*, and (**F**) *TSPO* in roots of *Arabidopsis* plants grown in ceramics soil. An asterisk above a column indicates that the value is significantly different from day 0 based on a t-test (p < 0.05). n = 3.

In *Arabidopsis*, previously generated microarray data related to drought stress response were analyzed in order to evaluate the expression of the *PDC* and *ALDH* gene family^3^ (Table 1). Among the 14 members of the *Arabidopsis* ALDH genes, expression of *ALDH2B7* was up-regulated during the 5^th^ to 9^th^ day in roots following a drought stress treatment, expression of *ALDH7B4* and *ALDH10A8* was up-regulated during the 7^th^ to 9^th^ day, and the expression of *ALDH2B4* was up-regulated only on the 9^th^ day. In shoots, the expression of *ALDH2B4*, *ALDH2B7*, and *ALDH7B4* was also significantly up-regulated in response to drought stress. ALDH2B4 and ALDH2B7 are predicted to localize to mitochondria, while ALDH7B4 is a cytosolic enzyme (Table 1). ALDH2B4 and ALDH2B7 are also phylogenetically related to each other; resulting in a subclade with ALDH2C4 (Fig. S1). The expression levels of *ALDH2B4* and *ALDH2B7* were both significantly up-regulated in root tissue by drought stress, however, the up-regulation of *ALDH2B7* occurred much earlier than *ALDH2B4*. RT-qPCR analysis further confirmed that *ALDH2B7* expression is significantly up-regulated in roots and shoots of WT plants in response to drought stress (Fig. 2).

**Table 1:** Expression profile of *ALDH* and *PDC* gene family members in *Arabidopsis*.

Gene	Name	Localization	Root (Fold change)				Shoot (Fold change)			
			3d	5d	7d	9d	3d	5d	7d	9d
***ALDH*** **Gene Family (Total 14 members in** ***Arabidopsis*** **)**										
*AT3G24503*	*ALDH2C4*	Cytoplasm^1^	1.56	1.09	1.00	1.74	1.01	1.00	1.00	1.00
*AT3G48000*	*ALDH2B4*	Mitochondria^1^	0.95	1.17	1.90	**3.45**	0.99	0.93	1.27	**2.53**
*AT1G23800*	*ALDH2B7*	Mitochondria^1^	1.47	**2.37**	**4.10**	**7.06**	1.12	1.23	1.58	**3.11**
*AT4G34240*	*ALDH3I1*	Chloroplast^1^	0.91	1.10	0.89	0.94	1.17	0.98	0.74	0.74
*AT1G44170*	*ALDH3H1*	Cytoplasm^1^	0.93	0.75	0.62	0.75	1.24	1.28	1.29	1.20
*AT4G36250*	*ALDH3F1*	Cytoplasm^1^	0.88	1.31	1.34	1.40	0.91	0.96	0.92	0.34
*AT1G79440*	*ALDH5F1*	Mitochondria^1^	0.87	0.87	0.99	1.33	1.09	1.05	0.86	0.90
*AT2G14170*	*ALDH6B2*	Mitochondria^1^	0.79	0.55	0.33	0.34	1.04	1.10	1.09	1.13
*AT1G54100*	*ALDH7B4*	Cytoplasm^1^	1.26	1.58	**2.13**	**3.34**	1.65	**2.77**	**9.11**	**23.96**
*AT1G74920*	*ALDH10A8*	Plastid^2^	1.05	1.69	**2.58**	**2.68**	1.08	1.07	1.18	1.07
*AT3G48170*	*ALDH10A9*	Peroxisomes^2^	0.99	1.11	0.95	1.21	1.06	1.02	1.02	1.27
*AT2G24270*	*ALDH11A3*	Cytoplasm^2^	1.13	1.01	1.02	0.99	0.75	0.78	0.81	0.74
*AT5G62530*	*ALDH12A1*	Mitochondria^1^	0.95	0.75	1.07	1.83	0.89	1.13	0.94	1.63
*AT3G66658*	*ALDH22A1*	Cytoplasm^1^	1.00	1.03	0.84	0.76	1.00	0.91	0.71	0.41
***PDC*** **Gene Family (4 members in** ***Arabidopsis*** **)**										
*AT4G33070*	*PDC1*	Cytoplasm^3^	1.74	**4.38**	**7.92**	**10.71**	1.19	1.16	**2.52**	**3.95**
*AT5G54960*	*PDC2*	Cytoplasm^3^	0.93	1.42	1.58	**2.14**	0.94	0.94	0.71	1.45
*AT5G01330*	*PDC3*	Plastid^3^	1.42	1.24	0.47	0.13	0.80	0.74	0.60	0.64
*AT5G01320*	*PDC4*	Cytoplasm^3^	1.00	1.07	0.77	0.70	0.93	0.95	0.76	0.57
***TSPO*** **Gene**										
*AT2G47770*	*TSPO*	Golgi network^4^	8.46	**57.68**	**167.7**	**364.6**	**6.73**	**31.34**	**861**	**1746**

^1^As reviewed^12^.

^2^As described previously^13^.

^3^*In silico* prediction as determined during current studies using SUBA–the Arabidopsis Subcellular Database: suba3.plantenergy.uwa.edu.au.

^4^As described previously^20,21^.

Microarray data (Rasheed *et al*.^3^) were analyzed to determine changes in the expression of *ALDH* and *PDC* family genes. Values shown in bold are significantly up-regulated according to FDR < 0.1, fold change >2.

Among the 4 members of *Arabidopsis* PDC family, expression of *PDC1* was significantly up-regulated in roots and shoots in response to drought stress, while the expression of *PDC2* in roots was only slightly up-regulated on the 9^th^ day after drought stress (Table 1). The expression of *TSPO* was similarly up-regulated in roots and shoots by drought stress (Table 1). RT-qPCR analysis further confirmed the up-regulation of *PDC1, ALDH2B7 *and *TSPO* in roots and shoots in response to drought stress (Fig. 2).

### GUS analysis of *TSPO* and *PDC1* promoter activity

**Figure 3:**
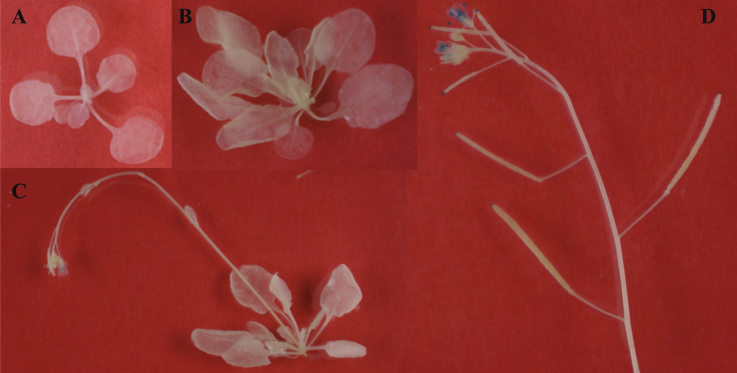
Changes in the expression of *TSPO* during different developmental stages of *Arabidopsis*. (**A**) 2, (**B**) 3, (**C**) 5, and (**D**) 7 weeks after sowing.

**Figure 4:**
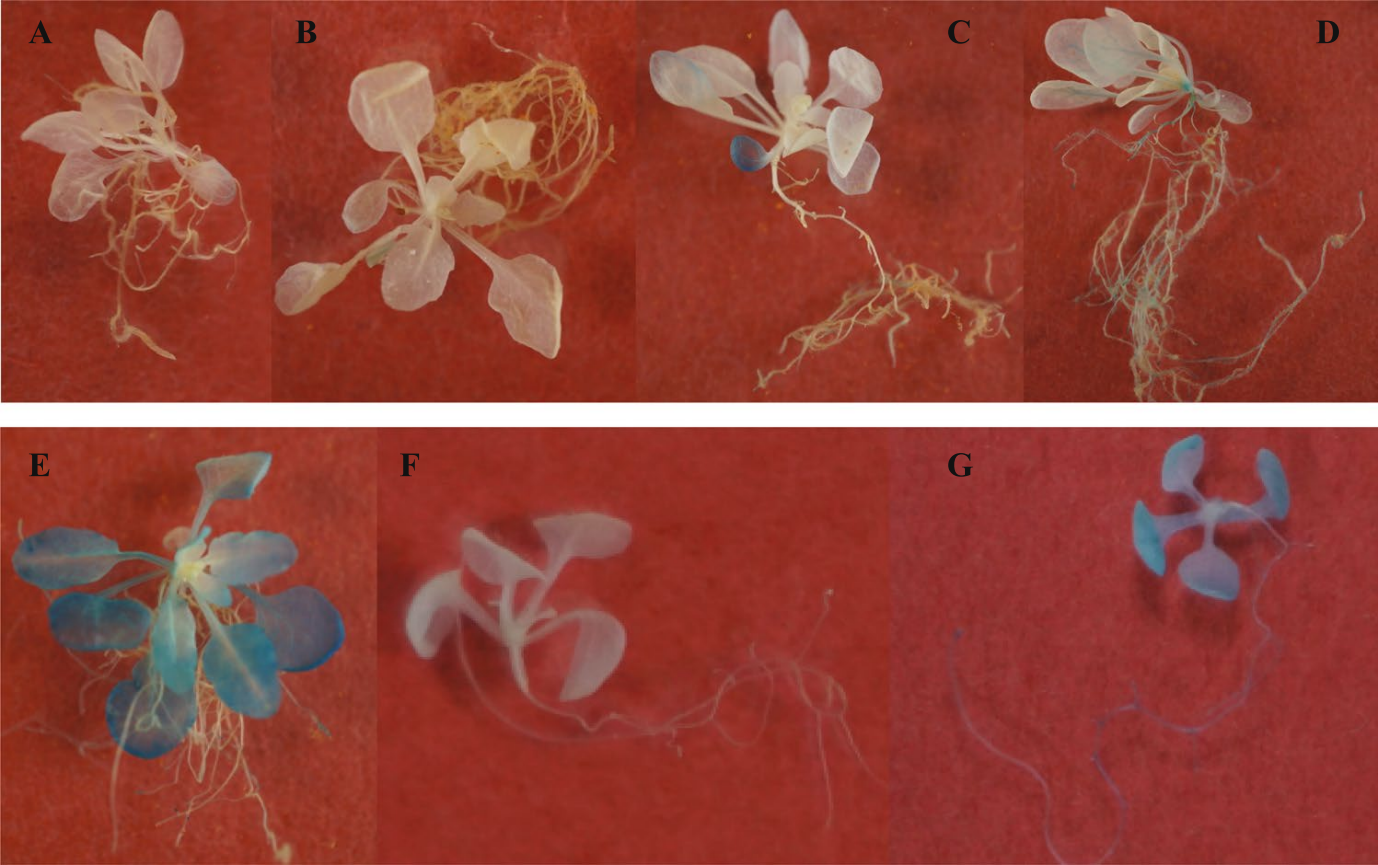
Analysis of *TSPO* and *PDC1* promoter driven *GUS* expression. (**A**–**E**) *TSPO* promoter driven *GUS* expression in plants grown in ceramic-based granular soil at (**A**) 0, (**B**) 1, (**C**) 3, (**D**) 5, and (**E**) 7 days after the initiation of the drought stress treatment. Plants were grown in a ceramic-based granular soil. (**F**,**G**) *PDC1* promoter of *GUS* expression in non-stressed and drought stressed plants grown on MS. (**F**) non-stressed, control plants (**G**) plants removed from the MS medium and subjected to 2 h of exposure to dry air in a laminar airflow cabinet.

In *Arabidopsis*, *TSPO* is a small, intronless gene located on chromosome 2 (Fig. S2A). In contrast, *PDC1* contains 5 exons, while *ALDH2B7* is composed of 11 exons (Fig. S2B,C). A 1.4 kb promoter sequence upstream of the *ATG* start codon of *TSPO* was used to drive the expression of *uidA* which allowed for subsequent analysis by GUS staining (Fig. S2D). Although the GUS expression driven by *TSPO* promoter was not detected during vegetative growth under normal growth conditions (Fig. 3A,B), analysis of four independent transgenic lines revealed expression during the early to late stages of flowering in anthers, ovaries, and the tips of siliques (Fig. 3C,D). GUS analysis of the *TSPO* promoter confirmed that *TSPO* expression is significantly up-regulated in roots and shoots beginning at day 3 of a drought stress treatment. Up to the 5^th^ day of the drought stress treatment, GUS staining was evident in young leaves and also surrounding the vascular tissue (Fig. 4A–D). GUS expression was visible in all leaves by the 7^th^ day of the drought stress treatment. GUS localization was also observed in root tissue, however, the GUS staining was weak in relative comparison to the staining in shoot tissue (Fig. 4C–E). These results demonstrated that the *TSPO* promoter is a good candidate to regulate the gene expression in a drought-specific manner.

GUS localization driven by the *PDC1* promoter was not observed in ten days-old plants growing on MS medium. In contrast, however, strong GUS localization was observed in roots and shoots two hours after plants were subjected to a dry air treatment by uprooting the plants and placing them in a laminar flow cabinet. These data indicate that the expression of *PDC1* is readily induced by drought stress (Fig. 4F,G).

### Localization of PDC1:GFP

**Figure 5:**
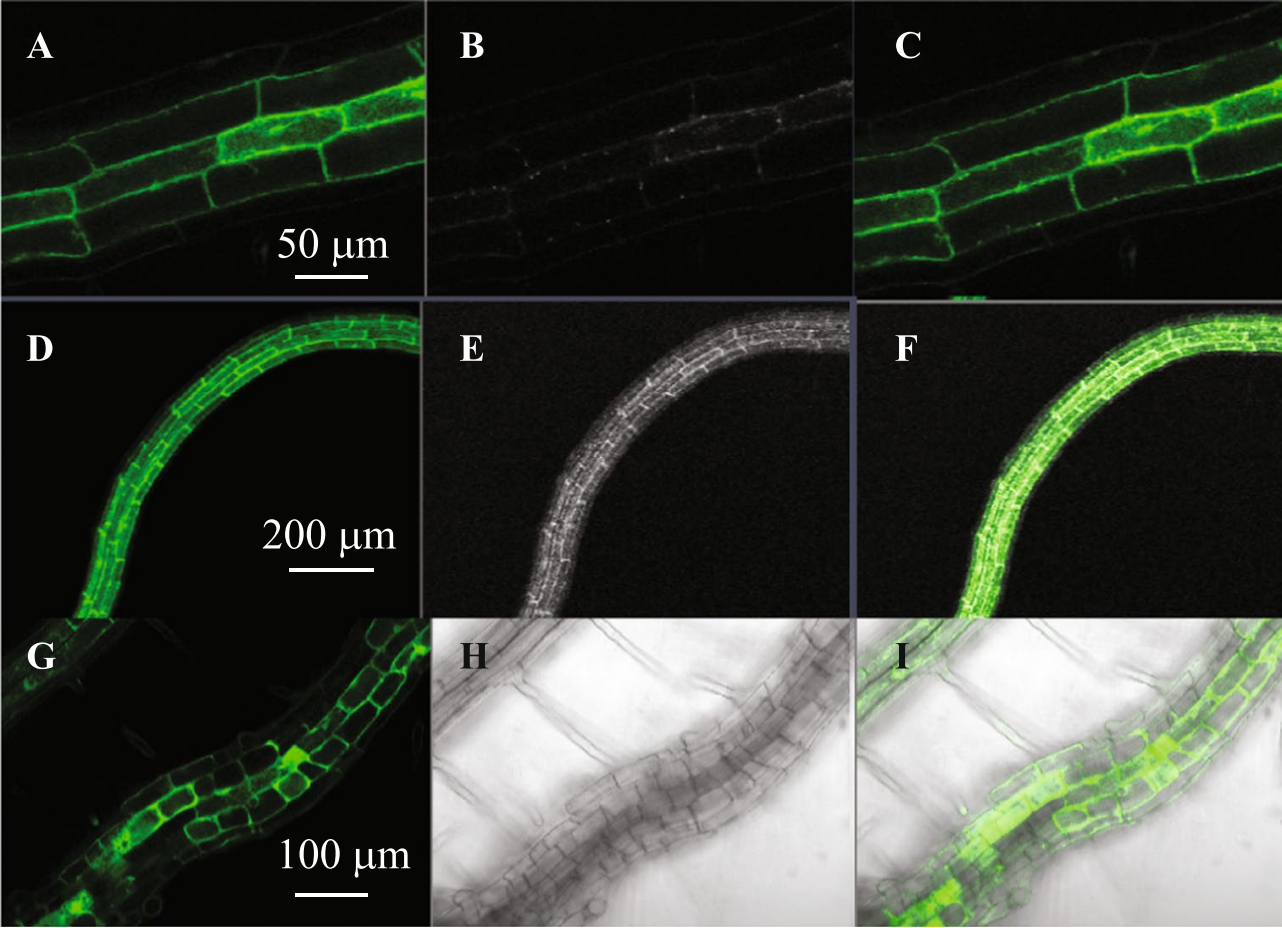
Localization of PDC1 in *Arabidopsis* roots. Localization of PDC1p:PDC1::GFP in *Arabidopsis* roots (**A**,**D**) GFP fluorescence, (**B**,**E**) transverse image, (**C**,**F**) image overlay of PDC1:GFP homozygous lines (**G**) GFP fluorescence, (**H**) transverse image, (**I**) image overlay of sGFP only lines.

*In silico* analysis indicated that PDC1 localizes to the cytoplasm, however, its localization has not been verified. Therefore, to confirm its localization, four independent transgenic lines expressing *PDC1* fused to *GFP* under the control of 1.9 kb *PDC1* promoter sequence were analyzed. Results confirmed that PDC1 localizes to the cytoplasm of *Arabidopsis* root cells (Fig. 5A–F). As shown in Table 1, ALDH2B7 is reported to localize to mitochondria^12^. These data suggest that PDC1 converts pyruvate into acetaldehyde in the cytoplasm, which may then be transferred to mitochondria where acetaldehyde is converted to acetate by ALDH2B7.

### *TSPO* promoter driven expression of *PDC1* and *ALDH2B7* in transgenic plants

**Figure 6:**
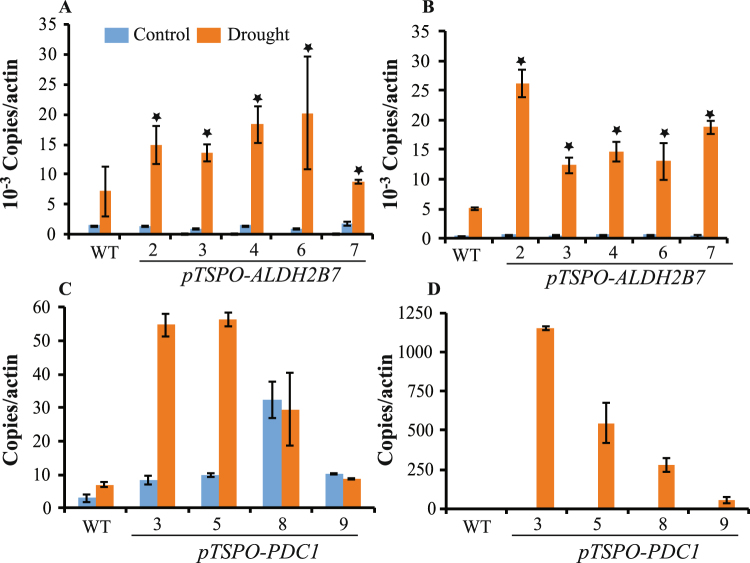
Confirmation of *PDC1* or *ALDH2B7* expression in transgenic lines of *Arabidopsis*. (**A**,**B**) Expression of *ALDH2B7* in transgenic lines (**A**) root and (**B**) shoots. (**C**–**D**) Expression of *PDC1* in transgenic lines (**C**) root and (**D**) shoots. An asterisk above a column indicates that the value is significantly different from WT plants based on a t-test (p < 0.05). n = 3.

To characterize the effect of the *TSPO* promoter *in planta*, a total of nine *pTSPO-PDC1* and seven *pTSPO-ALDH2B7* homozygous transgenic lines were developed. Initial evaluation of *PDC1* and *ALDH2B7* expression driven by the *TSPO* promoter, and drought tolerance in the transgenic lines, reduced the number to five *pTSPO-ALDH2B7* and four *pTSPO-PDC1* lines (Data not shown). Under normal growth conditions, the expression of *TSPO* is very low and the expression of both *PDC1* and *ALDH2B7* driven by the *TSPO* promoter was also low under normal growth conditions (Fig. 6A,B). After analyzing expression under normal growth conditions, the transgenic lines were subsequently grown in ceramic granular soil and subjected to a simulated drought stress. RT-qPCR analysis demonstrated that the expression of *PDC1* and *ALDH2B7* in the transgenic lines was specifically up-regulated by drought stress, relative to their expression in WT plants (Fig. 6).

In *pTSPO-ALDH2B7-2*, 3, 4, 6, and 7 transgenic lines, *ALDH2B7* expression in roots and shoots of lines was comparable to WT plants under normal, non-stress conditions. In contrast, the expression of *ALDH2B7* was significantly higher in the roots and shoots of these lines, relative to their expression in WT plants, seven days after the plants were subjected to drought stress (Fig. 6A,B). These results demonstrate that the *TSPO* promoter can be effectively used to regulate gene expression in a drought-specific manner.

The expression of *PDC1* was also significantly higher in *pTSPO-PDC1* transgenic lines (*pTSPO-PDC1-3*, *pTSPO-PDC1-5*, *pTSPO-PDC1-8*, *pTSPO-PDC1-9*), relative to WT plants, in response to drought stress. In these lines, the expression of *PDC1* in roots was also higher in transgenic plants compared to the expression of these genes in WT plants; even under non-stress conditions (Fig. 6C).

### *pTSPO-PDC1* or *pTSPO-ALDH2B7* transgenic plants exhibit higher survival ratio under drought stress conditions

**Figure 7:**
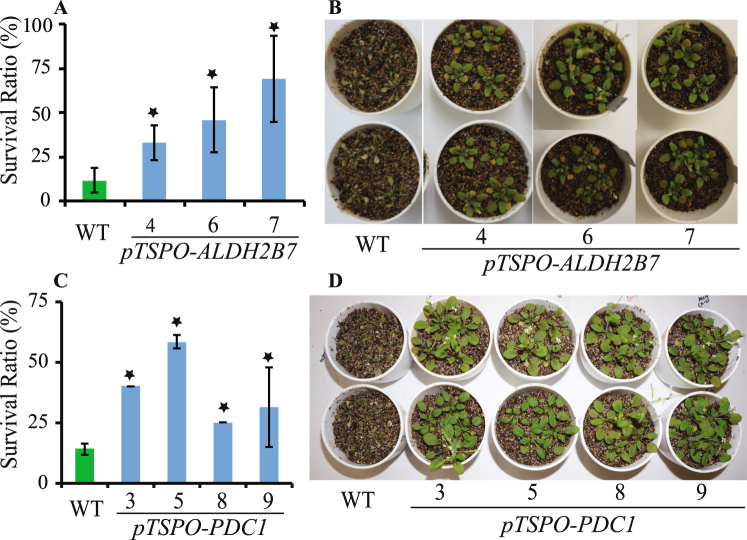
*pTSPO-ALDH2B7* or *pTSPO-PDC1* transgenic lines exhibit improved survival ratio under drought stress. (**A**) Survival ratio of *pTSPO-ALDH2B7* lines. (**B**) WT and transgenic plants were re-watered after exposure to 15 days of drought stress (water withheld). (**C**) Survival ratio of *pTSPO-PDC1* lines. (**D**) WT and transgenic plants were re-watered after exposure to 15 days of drought stress (water withheld). An asterisk above a column indicates that the value is significantly different from WT plants based on a t-test (p < 0.05). n = 4.

Different homozygous transgenic lines harboring *pTSPO-PDC1* or *pTSPO-ALDH2B7* were selected and subjected to a drought stress treatment. Three transgenic lines (*pTSPO-ALDH2B7-4*, *pTSPO-ALDH2B7-6*, and *pTSPO-ALDH2B7-7*) expressing *ALDH2B7* under the control of the *TSPO* promoter exhibited a significantly higher survival ratio, relative to WT plants, in response to the drought stress treatment (Fig. 7A,B). Similarly, transgenic lines expressing *pTSPO-PDC1* under control of the *TSPO* promoter also exhibited significantly higher survival ratio, relative to WT plants, under drought stress treatment (Fig. 7C,D). Importantly, no significant phenotypic changes, other than higher survival ratio under drought stress conditions, were observed in transgenic plants. The fresh weight of roots and shoots of plants expressing *PDC1* or *ALDH2B7* was comparable to WT plants when grown in ceramics granular soil (Fig. S3).

### Microarray Analysis

**Figure 8:**
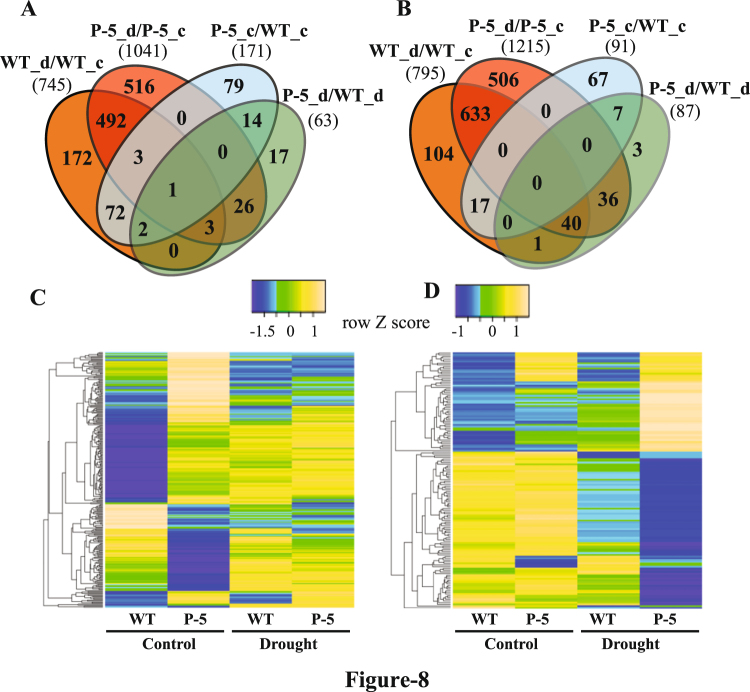
Summary of transcriptomic changes in WT and *pTSPO-PDC1-5* in response to drought stress. (**A**,**B**) Venn diagram summarizing the number of genes up- (**A**) or down-regulated (**B**) in response to drought stress. (**C**,**D**) Heat map analysis of genes with at least two-fold increase or decrease in expression in *pTSPO-PDC1-5* (*P-5*) plants compared to WT plants. (**C**) Genes differentially expressed in *P-5* compared to WT plants when grown under well-watered conditions. (**D**) Genes differentially expressed in *P-5* compared to WT plants when grown under drought stress conditions. (n = 3, FDR < 0.05, fold change > 2).

**Figure 9:**
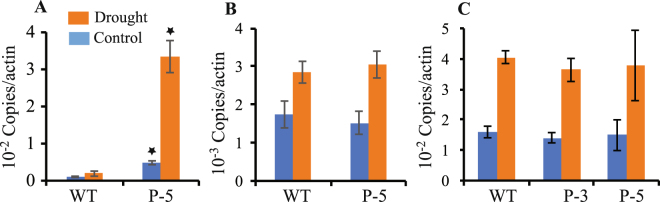
Confirmation of *PDC1, ADH1 and ALDH2B7* expression in WT and *pTSPO-PDC1* lines. Changes in the expression of *PDC1* (**A**), *ADH1* (**B**) and *ALDH2B7* (**C**) in WT and *pTSPO-PDC1* lines. Shoot samples were collected 11 days after drought stress treatment and plants that were maintained under watered conditions were used as controls. An asterisk above a column indicates that the value is significantly different from WT plants based on a t-test (p < 0.05). n = 3.

Microarray analysis was performed to reveal the transcriptomic changes in *pTSPO-PDC1* plants under control and drought stress conditions compared to WT plants. In WT plants, a total of 745 genes were significantly up-regulated in response to drought stress; among which, 499 genes were also up-regulated in *pTSPO-PDC1* (Fig. 8A). A total of 795 genes were down-regulated in WT plants under drought stress; among which, 673 were also down-regulated in *pTSPO-PDC1* plants under drought stress conditions (Fig. 8B). In *pTSPO-PDC1* plants grown under control conditions, 171 genes were significantly up-regulated compared to WT plants grown under similar conditions. Among these, 78 genes were also up-regulated in WT plants under drought stress conditions (Table S1). Heatmap analysis highlights the expression changes in WT and transgenic plants under control and drought stress conditions (Fig. 8C,D). A total of 63 genes were up-regulated in *pTSPO-PDC1* plants compared to WT plants under drought stress. In comparison to WT plants, the expression of *PDC1* was higher in *pTSPO-PDC1* plants under control and drought stress conditions (Table 2). These also included several genes that are involved in signaling (Such as *ERF71*) and are related to alternate respiratory pathways (such as *NDB3* and *NDB4*). The expression of the *Cox19-like CHCH family protein (AT5G09570)*, which is involved in positively regulating mitochondrial reactive oxygen response, was also significantly up-regulated^22^. Microarray analysis showed that *ADH1* and *ALDH2B7* expression levels are not up-regulated in *pTSPO-PDC1* plants in either control or drought stress conditions. These results were further confirmed through RT-qPCR analysis (Fig. 9). Mapman analysis indicated that the TCA cycle is regulated in *pTSPO-PDC1* lines to increase energy use efficiency under both control and drought stress conditions (Figs S4,S5). Gene ontology (GO) analysis also indicated that the expression of several catabolism related genes is altered in *pTSPO-PDC1-5* plants under control as well as drought stress conditions (Figs S4,S5). Although there is no clear direction for these metabolic changes, it seems that additional acetic acid could also be used as an energy source to conserve cellular energy. As a result, this would provide transgenic plants with an advantage during drought stress conditions.

**Table 2:** Genes significantly up-regulated in *pTSPO-PDC1* under drought stress compared to WT plants.

Gene	Name	Fold change		
		WT_D/WT_C	P5_C/WT_C	P5_D/WT_D
*AT4G33070*	*PDC1*	0.90	**17.36**	**31.35**
*AT5G54450*	*Hypothetical protein (DUF295)*	1.21	**31.75**	**25.97**
*AT2G03130*	*Ribosomal protein*	0.74	**9.13**	**14.01**
*AT2G47520*	*Ethylene Response Factor 71 (ERF71)*	0.86	**14.32**	**13.45**
*AT3G42658*	*SADHU3-2*	1.16	**14.53**	**8.13**
*AT5G09570*	*Cox19-like CHCH family protein*	**2.74**	**10.03**	**7.33**
*AT5G54420*	*Hypothetical protein (DUF295)*	0.97	**6.70**	**7.24**
*AT4G14060*	*Polyketide cyclase/dehydrase*	5.50	1.16	**6.89**
*AT2G20800*	*NAD(P)H dehydrogenase B4 (NDB4)*	0.82	**3.62**	**6.35**
*AT2G47760*	*Asparagine-linked glycosylation 3 (ALG3)*	0.93	**9.77**	**6.31**
*AT3G30720*	*Qua-Quine Starch (QQS)*	1.18	**6.61**	**5.84**
*AT2G01520*	*MLP-like protein 328 (MLP328)*	3.18	1.56	**5.35**
*AT1G18980*	*RmlC-like cupins superfamily*	0.70	0.65	**4.41**
*AT3G44042*	*SADHU3-1*	1.62	**9.39**	**4.35**
*AT5G26310*	*UGT72E3*	2.23	1.83	**4.13**
*AT5G17090*	*Cystatin/monellin superfamily protein*	1.44	1.46	**4.03**
*AT3G02590*	*Fatty acid hydroxylase*	1.29	1.00	**3.92**
*AT4G13280*	*Terpenoid Synthase 12 (TPS12)*	1.70	0.60	**3.70**
*AT1G58643*	*Inositol-penta kisphosphate 2-kinase family*	1.04	1.38	**3.63**
*AT5G51440*	*HSP20-like chaperones*	**2.59**	**6.79**	**3.50**
*AT3G01600*	*NAC044*	0.86	**3.58**	**3.29**
*AT4G06555*	*Hypothetical protein*	0.82	0.80	**3.25**
*AT3G15320*	*Transposable element*	0.91	1.78	**3.16**
*AT2G27550*	*Centroradialis (ATC)*	2.27	1.00	**3.10**
*AT2G21640*	*Unknown protein (marker of Oxidative stress)*	0.81	2.76	**3.09**
*AT1G35250*	*Acyl-Lipid Thioesterase 2 (ALT2)*	3.95	2.98	**3.04**
*AT1G19060*	*Hypothetical protein (DUF626)*	1.69	1.73	**2.81**
*AT5G38020*	*Methyltransferase*	**4.77**	1.32	**2.77**
*AT4G13290*	*CYP71A19*	2.10	1.36	**2.63**
*AT5G04370*	*NAMT1*	**2.54**	1.15	**2.62**
*AT1G14240*	*APYrase3 (APY3)*	1.63	1.04	**2.58**
*AT4G12470*	*Azelaic acid induced 1 (AZI1)*	**0.14**	0.64	**2.57**
*AT3G24750*	*LAZY5*	**14.60**	0.66	**2.55**
*AT4G26485*	*Methyltransferase*	1.22	0.76	**2.55**
*AT5G43340*	*Phosphate Transporter 1;6 (PHT1;6)*	1.68	1.01	**2.50**
*AT3G24230*	*Pectate lyase*	2.42	2.04	**2.47**
*AT1G26680*	*B3 family TF*	1.59	1.17	**2.44**
*AT1G19230*	*Riboflavin synthase-like*	2.46	1.78	**2.44**
*AT4G21490*	*NAD(P)H dehydrogenase B3 (NDB3)*	**3.52**	**3.90**	**2.41**
*AT2G19200*	*Hypothetical protein (DUF626)*	1.09	1.19	**2.40**
*AT5G64000*	*SAL2*	**0.05**	1.47	**2.38**
*AT3G26830*	*Phytoalexin Deficient 3 (PAD3)*	**0.07**	4.79	**2.37**
*AT4G12500*	*Seed storage 2S albumin*	**0.02**	0.50	**2.37**
*AT3G17010*	*Reproductive meristem 22 (REM22)*	1.30	1.37	**2.34**
*AT4G27570*	*UGT79B3*	1.27	1.62	**2.31**
*AT4G28790*	*bHLH DNA-binding superfamily*	1.32	1.01	**2.30**
*AT1G48180*	*Target of trans acting-siR480/255 protein*	0.95	1.07	**2.30**
*AT4G27980*	*Trichohyalin-like protein*	1.93	2.04	**2.28**
*AT5G50770*	*Hydroxysteroid dehydrogenase 6 (HSD6)*	0.98	1.01	**2.25**
*AT2G18193*	*Nucleoside triphosphate hydrolases*	1.06	**4.43**	**2.23**
*AT4G00250*	*ATSTKL2*	1.59	1.10	**2.21**
*AT1G71200*	*bHLH superfamily protein*	1.11	1.80	**2.20**
*AT3G10790*	*F-box associated ubiquitination effector*	1.15	1.00	**2.20**
*AT2G17950*	*WUSCHEL (WUS)*	3.82	1.49	**2.19**
*AT5G24640*	*Hypothetical protein*	1.20	**3.13**	**2.17**
*AT1G54590*	*Pre-mRNA-splicing factor-like protein*	1.49	2.42	**2.15**
*AT3G12450*	*Pseudogene of 3′-5′ exonuclease*	2.33	1.00	**2.15**
*AT5G47600*	*HSP20-like chaperones*	1.75	1.35	**2.13**
*AT2G18150*	*Peroxidase superfamily protein*	1.65	1.04	**2.10**
*AT1G46552*	*Transposable element*	1.75	1.84	**2.09**
*AT3G25010*	*Receptor like protein 41 (RLP41)*	**0.09**	2.02	**2.07**
*AT3G02885*	*GAST1 protein homolog 5 (GASA5)*	1.88	0.83	**2.04**
*AT1G71690*	*Glucuronoxylan 4-O-methyltransferase-like*	1.72	1.18	**2.03**

Values shown in bold are significantly up or down-regulated according to FDR < 0.05, fold change > 2.

The English in this document has been checked by the professional language editors.

## Discussion

Drought is a serious agricultural problem, and therefore, the development of drought tolerant plants is a very important technology that would allow us to better ensure our ability to meet the increasing demand of food as the population of the world increases^1^. Several strategies have been used to engineer transgenic plants that can tolerate low water availability, including the overexpression of late-embryogenesis-abundant (LEA) proteins, osmolyte biosynthesis genes, transcription factors and kinases, and ABA-signaling related genes. All of these approaches have resulted in the creation of transgenic plants with varying levels of increased drought tolerance^1,23–29^. Transgenic maize lines have been tested in field conditions where they offered several advantages compared to WT plants under water limiting conditions^30–32^. In this regard, the molecular breeding of drought tolerant crops that utilize improved strategies could potentially alleviate the adverse effects of overexpressing specific genes to improve drought tolerance. The use of a suitable promoter to regulate the expression of genes that contribute to drought tolerance is essential in order to avoid any undesirable impacts on plant growth and yield^16^. Since roots sense water scarcity much earlier than shoots, an ideal promoter would first up-regulate target genes in roots and specifically during the early phase of drought stress.

In the present study, in order to identify a suitable promoter^3^, previously generated microarray data were used to analyze changes in the expression of genes in roots and shoots in soil-grown plants subjected to a drought stress. The analysis indicated that the *Arabidopsis TSPO* gene exhibited a basal, low-level of expression under non-stress conditions and was significantly up-regulated in roots and shoots during the early phase of drought stress (Table 1). RT-qPCR analysis further confirmed the expression pattern of *TSPO* in *Arabidopsis* roots and shoots. Promoter-*GUS* analysis also demonstrated that *TSPO* expression is very low under normal growth conditions. During the very early stage of drought stress response, *TPSO* is up-regulated and increases in expression as drought stress progresses. In *Arabidopsis*, TSPO has been reported to bind to the plasma membrane aquaporin (PIP2;7), as well as endoplasmic reticulum and Golgi membranes where it regulates the cell-surface expression of PIP2;7 under abiotic stress conditions^20^. The strict up-regulation of *TSPO* by ABA treatment^21^ and drought stress suggested that the *TSPO* promoter would be a good candidate to regulate the expression of transgenes in a drought responsive manner. The acetic acid pathway genes, *PDC1* and *ALDH2B7*, were then selected and placed under the control of the TSPO promoter to determine if they would enhance drought tolerance in transgenic *A*r*abidopsis* plants in a controlled manner.

PDC and ALDH family members play a functional role in the biosynthesis of acetic acid in plants^7^. The *PDC* gene family is confined to plants and fungi, while the *ALDH* family is ubiquitous in yeast, plants, and animals^11,33–35^. *PDC1* transcripts comprise approximately 10–40% of the total *PDC* mRNA in seedlings under normal growth conditions, while this proportion reaches up to 99% under oxygen limiting conditions^9^. ABA and mannitol treatments also up-regulate the expression of *PDC1* and *ALDH2B7*^9^. Indeed the whole ethanol-acetic acid pathway is up-regulated by oxygen limiting conditions and ABA treatment, as well as under drought stress conditions^3,9,36^. *PDC1* is expressed more highly in siliques and seeds^9^. In plants, members of *ALDH2* family detoxify acetaldehyde generated during ethanol fermentation by converting it to acetate^8,11,37^. Acetate is further converted to acetyl-CoA via acetyl-CoA synthase activity. In *Arabidopsis*, 14 members of ALDH family have been described^12^ (Table 1 & Fig. S1). ALDH2B7 is phylogenetically related to ALDH2B4 and ALDH2C4 (Fig. S1). Characterization of mutants of the *Arabidopsis ALDH* family have revealed functions associated to drought stress. In the *aldh2B7* mutant, the conversion of ethanol to fatty acids is not affected, while conversion is significantly decreased in the *aldh2B4* single and *aldh2B4*/*aldh2B7* double mutants. Thus, under normal growth conditions, ALDH2B4 has been suggested to play a dominant role in the PDH bypass pathway^8^. The expression of *ALDH2B7* in response to drought stress is up-regulated much earlier than *ALDH2B4* (Table 1), suggesting that the role of ALDH2B7 in the response to drought stress may be more critical than ALDH2B4.

Similarly, among the *Arabidopsis* PDC members, the expression of *PDC1* is more highly up-regulated by drought stress than *PDC2*, *PDC3*, and *PDC4* (Table 1). The expression of *PDC1* was observed during different developmental stages of *Arabidopsis* (Fig. S6). As previously mentioned, *Arabidopsis PDC1* and *ALDH2B7* genes are involved in the acetic acid fermentation pathway (Fig. 1) and the expression of *ALDH2B7* is up-regulated in response to ABA application and osmotic stress. Similar to *PDC1*, the expression of *ALDH2B7* was also observed during different developmental stages of Arabidopsis (Fig. S7). The current study confirmed that the expression of *ALDH2B7* is up-regulated in both roots and shoots by drought stress. A previous study suggested that ALDH may play a role in the aerobic detoxification of acetaldehyde and that ALDH activity during stress might occur independently of ethanol fermentation^9^. The role of *Arabidopsis PDC* genes in ethanol fermentation has also been previously described^10^, however, its role in the production of acetate through the PDH independent pathway has not been described in detail. The collective analysis of the published data clearly indicates that these genes could play an important role in drought stress response through a PDH independent pathway. It is plausible that the continuous production of acetic acid could have a negative impact on plant growth. This was experimentally verified in the current study by watering two-week-old *Arabidopsis* plants once a week for five weeks with 10 mM acetic acid. Plants treated with acetic acid were dwarfed and produced less biomass relative to control plants (Fig. S8). These results indicated that selecting a suitable promoter, such as the *TSPO* promoter, would be extremely important to the development of drought stress tolerant plants while avoiding any undesirable effects on growth and yield. Microarray data also suggested that plants expressing *ALDH2B7* and *PDC1* under the control of the TSPO promoter could provide significant tolerance to drought stress. Therefore, transgenic *Arabidopsis* plants expressing either *PDC1* or *ALDH2B7* under the control of the drought stress-inducible *TSPO* promoter were developed to test this hypothesis.

A total of 7 and 9 homozygous transgenic lines were developed for *pTSPO-PDC1* and *pTSPO-ALDH2B7*, respectively. RT-qPCR analysis confirmed that the level of expression of *PDC1*and *ALDH2B7* under normal growth conditions was comparable to WT plants. In addition, in relative comparison to WT plants, the expression of *PDC1* and *ALDH2B7* in the transgenic plants was specifically up-regulated by drought stress. Three independent transgenic lines of *pTSPO-ALDH2B7* and four lines harboring *pTSPO-PDC1* exhibited significantly higher survival ratio under drought stress. These results confirmed our hypothesis that the regulation of acetic acid biosynthesis pathway genes could significantly enhance survival ability of plants under drought stress. Since the modulation of the acetic acid pathway via the constitutive overexpression of *PDC1* and *ALDH2B7* could negatively impact plant growth, the use of the *TPSO* promoter, which is specifically regulated by drought stress, can minimize off-types by driving expression only during periods of stress exposure. Regulating drought stress tolerance by modulating the expression of acetic acid pathway genes is a unique strategy. Although transgenic plants expressing *pTSPO-ALDH2B7* or *pTSPO-PDC1* have not been previously evaluated for drought stress tolerance, plants expressing *PDC* or *ALDH* family members have been tested for their response to other abiotic stresses^8–10,15,38,39^. PDC1, which is localized in the cytoplasm, converts pyruvate into acetaldehyde which may then be transferred to mitochondria where it is converted to acetate by ALDH2B7 in a well-coordinated manner. The development and analysis of transgenic plants exhibiting improved survival ratio under drought stress by modulating the expression of acetic acid pathway genes increases our understanding of the mechanisms responsible for drought stress tolerance in plants and also provides an opportunity to use this approach to ameliorate the injury caused by drought stress in field-grown crops. In a previous study, treatment of plants with acetic acid did not confer any undesirable phenotype in roots and shoots^5^. Similarly, the transgenic plants generated in the present study also did not exhibit any undesirable phenotypes. As a result, these observations confirm that the *TSPO* promoter is a good tool that can be used to regulate the expression of genes in a drought-responsive manner.

It is possible that the PDH bypass contributes to maintaining an optimal metabolic rate when plants are subjected to abiotic stresses and therefore minimizes the deleterious impact that abiotic stress has on plants. Acetyl-CoA plays a role in fatty acid metabolism. Exogenous application of labelled acetate and ethanol are readily incorporated into fatty acids and approximately 90% of this incorporation relies on the activity of the ACS enzyme^7^. Specific plant tissues, such as floral tissues, have been reported to undergo aerobic fermentation^37,40–42^, where pyruvate is converted to ethanol or acetate^7^. Subsequently, ACS converts the acetate to acetyl-CoA for use in fatty acid biosynthesis (Fig. 1). The importance of this pathway during flowering has been reported, however, it is not clear if acetate is processed in the same manner during drought stress. Importantly, the expression of *ADH* is significantly up-regulated by drought stress; however, the expression of mitochondrial components of *PDH* are significantly down-regulated (Fig. 1) by drought stress^3^. These observations indicate that the role of the PDH bypass pathway during drought response is extremely important for the synthesis of acetic acid, as well as acetyl-CoA. In addition to participating in fatty acid metabolism as part of a stress response^7,43^, the acetate produced by the enhanced expression of *PDC1* or *ALDH2B7* could also participate in histone acetylation, where the acetyl functional group of acetyl-CoA is transferred to histone^44^. Histone acetylation is an important aspect of the epigenetic regulation of gene expression in eukaryotes. In this process, acetyl-CoA acts as a metabolic signal regulating plant response to various abiotic stresses, as well as normal growth, through epigenetic changes involving histone acetylation^44–47^. Acetic acid enhances drought stress tolerance through jasmonate synthesis and enrichment of histone H4 acetylation; and by contributing to the induction of the jasmonate signaling pathway^5^. Acetic acid activates the COI1-mediated signaling pathway and the transient production of JA may define which downstream target genes are activated^5^ to enhance drought tolerance.

Microarray analysis revealed the up- and down-regulation of several genes related to primary and secondary metabolism (Figs S4 and S5). The expression of *ADH1* is not significantly altered, which indicates that ethanol synthesis may not be related to drought stress tolerance in *pTSPO-PDC1* lines. Although the expression of *ALDH2B7* is also not significantly up-regulated in *pTSPO-PDC1*, the overlapping phenotype of *pTSPO-PDC1* and *pTSPO-ALDH2B7*, as well as the exhibition of drought tolerance by the external application of acetic acid, supports the claim that acetic acid could increase drought stress tolerance in plants. Several signaling related genes, such as *ERF71*, were also up-regulated in *pTSPO-PDC1* plants compared to WT plants. *ERF71* mediates osmotic and hypoxia stress response in *Arabidopsis*^48^. In *Arabidopsis*, NAD(P)H dehydrogenases are located on both sides of the inner mitochondrial membrane as part of the non-phosphorylating alternative pathway and play important roles in the development of the plant and its ability to cope with oxidative stress^49^. The expression of two members of NAD(P)H dehydrogenases (*NDB3*, *NDB4*) which face the intermembrane space^50^ was significantly up-regulated in *pTSPO-PDC1* plants compared to WT plants, which suggests that transgenic plants are using alternative respiratory pathways. The changes in expression of other genes (Table 2, Table S1 & Figs S4 and 5) also suggest that *pTSPO-PDC1* plants might be selectively regulating the metabolism using alternate energy pathways to conserve energy which gives them an advantage under drought stress conditions. GO analysis also highlights that the expression of several metabolic related genes was significantly altered in *pTSPO-PDC1* compared to WT plants. Thus, in transgenic plants, in addition to JA signaling, metabolism is also regulated; which may help to conserve energy and thus provide an advantage to confer drought stress tolerance. It should be noted, however, that acetic acid mediated drought stress tolerance is a novel mechanism and further studies (such as NMR analysis) are needed to decide the fate of acetic acid in this energy conservation pathway.

In summary, the analysis of previously-generated microarray data resulted in the identification of the *TSPO* promoter that can be used to specifically up-regulate genes in both roots and shoots starting in the early phase of the response to drought stress. Transgenic plants expressing the acetic acid biosynthesis pathway genes, *PDC1* or *ALDH2B7*, under the control of the TSPO promoter exhibited phenotypes with enhanced survival ratio under drought stress conditions. These results demonstrate that modulating the acetic acid biosynthesis pathway can be a useful strategy for the development of plants with the ability to survive longer under drought stress conditions. In current studies, we have developed and analyzed *Arabidopsis* plants which exhibit ability to survive under drought stress conditions. Since external application of acetic acid has been shown to confer drought stress tolerance in rapeseed, maize, rice and wheat plants in addition to *Arabidopsis*^5^, it is reasonable to propose that this transgenic technology may also be effective in other crop plants.

## Materials and Methods

### Development of transgenic lines

A 1.4-kb region upstream of the ATG start site of the *TSPO* gene in *Arabidopsis* was cloned using the forward and reverse primers, 5′-ATAGTAGACTGCGGCCGCACCATGGCA-3′ and 5′-TGTCCTGAGATCTAGATACAAACGAAACGTCCAAAAC-3′. A *Not*I site was introduced into the forward primer, and a *Xba*I site was introduced into the reverse primer. The obtained *TSPO* promoter was then cloned into the pGreenII 0029 and pGreenII 0229 vectors. *ALDH2B7* and *PDC1* genes were cloned downstream of the *TSPO* promoter in the pGreenII 0229 and the pGreenII 0029 vectors, respectively. The primers used for the cloning of *PDC1* were 5′-CTCGAATTCCCCGGGTTAATTAAATTAATC-3′ and 5′-ATCAGGCCGTCGACGCCGGATC-3′ with introduced *Sma*I and *Sal*I sites, respectively. The primers used for the cloning of *ALDH2B7* were 5′-AATCGGCCCCCGGGTTAATTAAATTAATC -3′and 5′-TGGAGCTAGTCGACTTATGGCCGA-3′ and also contained introduced *Sma*I and *Sal*I sites, respectively. Transgenic *Arabidopsis* plants expressing either *pTSPO-ALDH2B7* or *pTSPO*-*PDC1* were created using *Agrobacterium tumefaciens* (GV3101::pMP90)-mediated transformation and the floral dip method^51^. Neomycin phosphotransferase II (NPT II) and the bialaphos resistance gene (bar) were part of the generated constructs and used to select for transformed plants using kanamycin (km) and bialaphos for *pTSPO-PDC1* and *pTSPO-ALDH2B7*, respectively.

In order to conduct a GUS analysis of the *PDC1* promoter, a 1.9 kb region upstream of the *PDC1* gene was amplified using the primers, 5′-CACCGACGCACTTTATTGTGTTGCAA-3′ and 5′-GGAAATGGGAAGGTTTGAGTTC-3′. The PCR product was cloned into the *pENTR/D-TOPO* (Invitrogen) vector. After sequence confirmation, the *PDC1* promoter was subcloned into *pMDC163* and expressed as the construct *PDC1p::uidA*. A similar approach was used for the GFP analysis, with an exception. Specifically, after sequence confirmation, the promoter construct was cloned into pMDC111 where it was used to drive expression of the *GFP* gene. Transgenic *Arabidopsis* plants expressing either the *PDC1* promoter fused to the GUS gene or the *PDC1* promoter fused to the *GFP* gene were created using *Agrobacterium tumefaciens* (GV3101)-mediated transformation with the floral dip method^51^. The vectors used for transformation are illustrated in Fig. S2D–H.

The gene sequences for *TSPO*, *PDC1*, and *ALDH2B7* (Fig. S2A–C) were acquired from the TAIR website (https://www.arabidopsis.org/). The hygromycin phosphotransferase gene was included in the construct that was used to transform *Arabidopsis* plants with *PDC1p::uidA*, and *PDC1p:PDC1::GFP* and Hygromycin B was used to select successfully transformed plants. Homozygous transgenic lines were developed and used in the subsequent experiments.

### Drought stress treatment

WT and transgenic seeds were sown in trays containing Dio propagation mix no. 2 professional soil (Dio Chemicals, Japan) and germinated and grown at 22 °C under a 16 h light/8 h dark cycle, and a ~100 µmol m^−2^ s^−1^ photon flux density. Two weeks after germination, plants were subjected to drought treatment by removing excess water from the trays and ceasing any subsequent watering. For microarray analyses, shoot samples were collected 11 days after drought stress treatment and plants that were maintained under watered conditions were used as controls. Samples were immediately frozen in liquid nitrogen and stored at −80 degrees until RNA extraction. Drought tests were performed with four replications and were repeated at least two times. Each replication consisted of 24 individual plants for WT and each transgenic line. In cases where the plants were grown in ceramic-based granular soil, the drought stress was applied as previously described^3^. Briefly, plants were grown on MS medium for 10 days at 22 °C and subsequently transferred to ceramic-based granular soil (size 2.5 L, Sakatanotane, Japan). After transfer to this substrate, plants were grown for an additional 7 days at 22 °C. The drought treatment was then administered by removing excess water from the trays and ceasing any subsequent watering. The survival ratio of WT and transgenic plants was calculated by counting the number of plants that survived at 24 and 48 hours after re-watering, relative to the initial number of total plants subjected to the drought treatment. Plants were harvested at 0 and 7 days after onset of the drought treatment and root and shoot fresh weights were measured.

### RT-qPCR analysis

Plants from lines that were homozygous for the *pTSPO-ALDH2B7* or *pTSPO-PDC1* genes were developed and drought-inducible expression was confirmed by RT-qPCR for the *ALDH2B7, PDC1*, and *TSPO* genes. cDNA from each sample was synthesized from 200 ng of extracted RNA using a QuantiTect Reverse transcription kit (QIAGEN, USA) according to the manufacturer’s instructions. The forward and reverse primers used for *PDC1* were 5′-AGAGCACCATCCTCATTTCATT-3′ and 5′-TGGCTTTTTCTTTCTTGAGGAG-3′, respectively. The forward and reverse primers used for *ALDH2B7* were 5′-CATGCTCATAGCAACAGACGA-3′ and 5′-CAGCAGCTAAACCGTACCTTG-3′, respectively. The forward and reverse primers used for *TSPO* were 5′-CTCAGTTTTTGCTCTGTTTGGTT-3′ and 5′-CAAACACGGCTTTACCAGATTAC-3′, respectively. For *ADH1*, the forward and reverse primers were 5′-AGACGATGCCTTCAAGACTCA-3′ and 5′-GGGAATGTCAGTTTTGGGTTT-3′, respectively. *Actin 2* was used as an internal control and amplified using the forward and reverse primers, 5′-TGAAGTGTGATGTGGATATCAGG-3′ and 5′-GATTTCTTTGCTCATACGGTCAG-3′, respectively. Changes in expression were recorded according to the standard curve method.

### Microarray analysis

Microarray analyses were carried out as described previously^3^ with few modifications. Total RNA (400 ng) was labeled with fluorescently-labeled Cy3, using a Quick Amp labeling kit (Agilent Technologies) and resulting cRNA was subsequently hybridized to an Agilent *Arabidopsis* custom microarray (GPL19830). Microarray analyses were performed with three biological replications. Arrays were scanned with a microarray scanner (G2505B, Agilent Technologies) and microarray data were processed and analyzed using GeneSpring GX (v.14.9, Agilent Technologies) with quantile normalization and median transformation. Statistical significance was assessed using a one way ANOVA with BH correction^52^ and a 95% confidence interval (Corrected *P*-value < 0.05). A Tukey’s HSD (honest significant difference) test with BH correction was performed as a post hoc test (Corrected *P*-value < 0.05). Genes with false discovery rate (FDR) values less than 0.05 and at least two-fold changes were regarded as up- or down-regulated. Heat map analyses were performed with an online tool heatmapper^53^. The normalized log_2_ values were then used to compare the transcriptomic changes using MapMan 3.6.0RC1^54^. Gene ontology analyses were carried out using the PANTHER (protein annotation through evolutionary relationship) classification system database maintained at http://pantherdb.org/ ^55^. The microarray data has been deposited to GenBank with the accession number GSE110857.

### *TSPO* and *PDC1* promoter driven GUS analysis

Transgenic plants derived from five homozygous lines of plants transformed with the *GUS* gene driven by the *TSPO* or *PDC1* promoter were used to analyze β-glucuronidase (GUS) staining. Plants used to evaluate *TSPO* driven *GUS* expression were grown in ceramic-based granular soil and samples were collected at 0, 1, 3, 5 and 7 days after the application of the drought treatment as previously described. *GUS* expression was observed in four independent transgenic lines. Plants used to analyze *PDC1* promoter activity were grown on MS medium for 10 days and samples were collected at 0 and 2 h after a drought treatment was administered. The drought treatment was initiated by pulling plants from MS medium and placing them in a petri dish which was subjected to the airflow of a laminar hood. The GUS analysis was conducted by dipping the plants twice in phosphoric acid buffer and then incubating them overnight in 0.5 mM X-Gluc (10 mM EDTA, 0.1% Triton X-100, 50 mM phosphoric acid buffer (pH 7.0), 0.5 mM potassium ferrocyanide, 2% DMSO) at 37 °C. The solution was then replaced with serial dilutions of ethanol and the samples were subsequently stored in 100% ethanol until they were photographed.

### Confirmation of the subcellular localization of PDC1

The cellular localization of PDC1, PDC2, PDC3 and PDC4 was predicted *in silico* using SUBA (*Arabidopsis* Subcellular Database) which is maintained at www.suba3.plantenergy.uwa.edu.au. The subcellular localization of PDC1 was also verified experimentally. Four independent homozygous lines of *pPDC1:PDC1::GFP* were examined in order to observe GFP localization in *Arabidopsis* roots using a LSM700 Laser scanning microscope with a 40 × Plan-Apochromat lens. A diode laser with a 488 nm excitation in conjunction with 488–555 nm and 560–700 nm) filters were used to observe the fluorescence of GFP. The obtained data were analyzed using ZEN 2011 software (ZEISS, Oberkochen, Germany).

### Phylogenetic Analysis

Protein sequences were acquired from The Arabidopsis Information Resource (TAIR) database and aligned by ClustalW in a CLC sequence viewer 8.0 (QIAGEN, Denmark). Subsequently, a phylogeny was constructed using a neighbor-joining (NJ) algorithm with the Jukes-Cantor distance measurement method^56,57^. The bootstrap analysis was carried out for the branching arrangements with 1000 replicates^58^.

## Electronic supplementary material

Supplemental Figures
