# Supplementary material for: The modulation of acetic acid pathway genes in Arabidopsis improves survival under drought stress
Source: Sci Rep. 2018 May 18;8:7831. doi: 10.1038/s41598-018-26103-2 (PMC5959891; doi:10.1038/s41598-018-26103-2)
Supplement: Supplementary file 1 — Supplemental Figures [file 41598_2018_26103_MOESM1_ESM.pdf]

The modulation of acetic acid pathway genes in *Arabidopsis* improves survival under drought stress

Sultana Rasheed, Khurram Bashir, Jong-Myong Kim, Marina Ando, Maho Tanaka, Motoaki Seki.

Scientific Reports, 2018

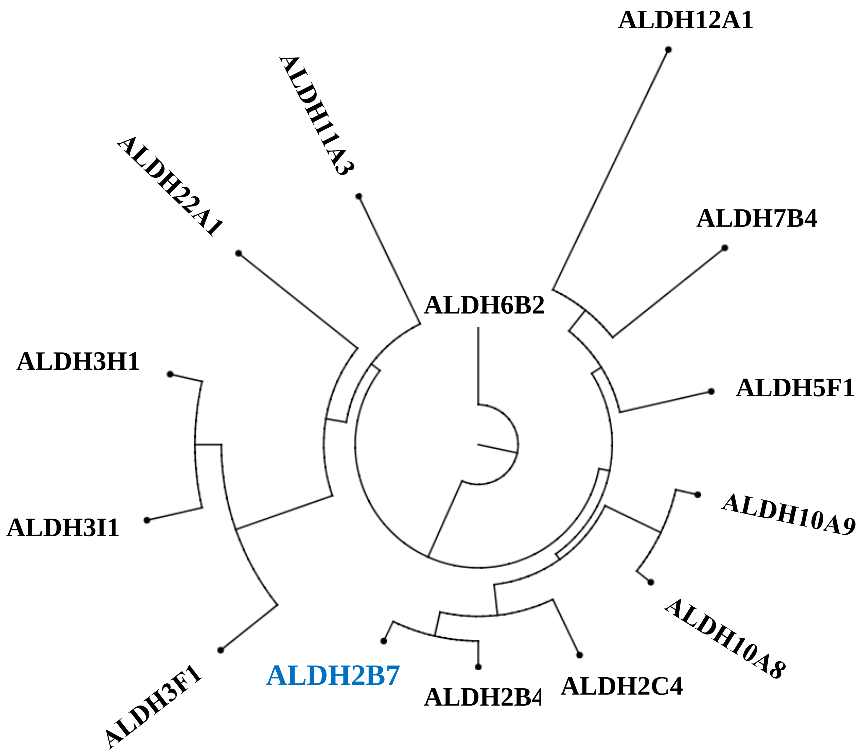

Figure-S1: Phylogeny of Aldehyde dehydrogenases in *Arabidopsis thaliana*

The accession numbers of ALDH family members in *Arabidopsis* are shown in Table-1. The protein sequences were acquired from TAIR database maintained at https://www.arabidopsis.org/.

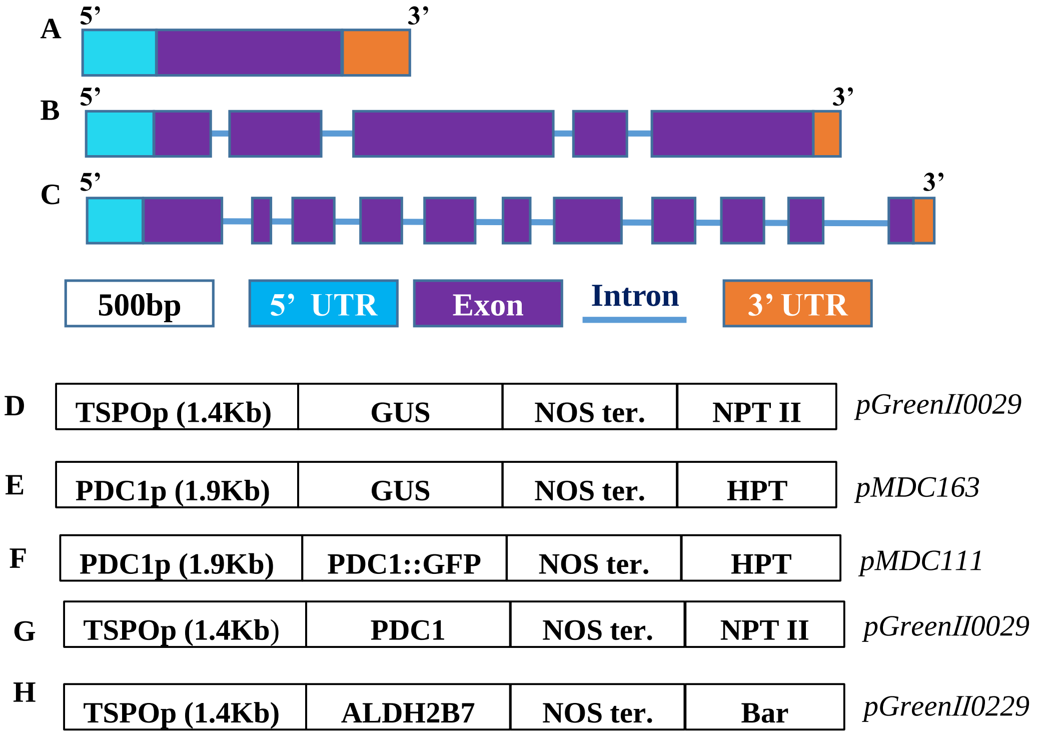

Figure-S2: Gene structure and vectors used for the cloning of *TSPO*, *PDC1* and *ALDH2B7*.
Gene structure of (**A**): *TSPO*, (**B**): *PDC1* and (**C**): *ALDH2B7*. Scale bar shows gene size for **A-C**. (**D-H**). Vectors used for the transformation of *Arabidopsis*.

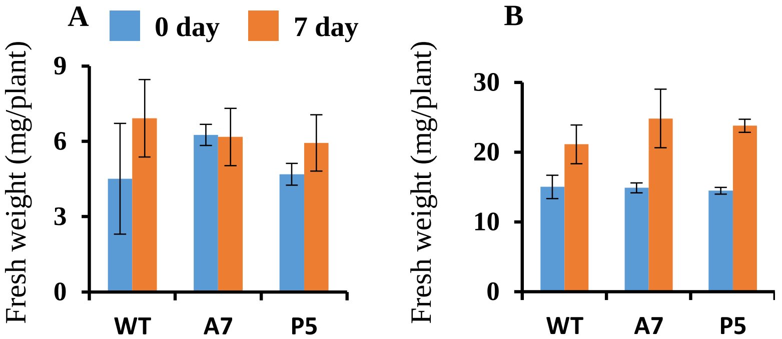

Figure-S3: Shoot and root fresh weight of WT and transgenic plants grown in ceramics granular soil

(**A**): Roots fresh weight of WT and transgenic lines. (**B**) Shoots fresh weight of WT and transgenic lines. The column bars followed by asterisk are significantly different from respective WT plants at given time point ($p<0.05$). n=5.

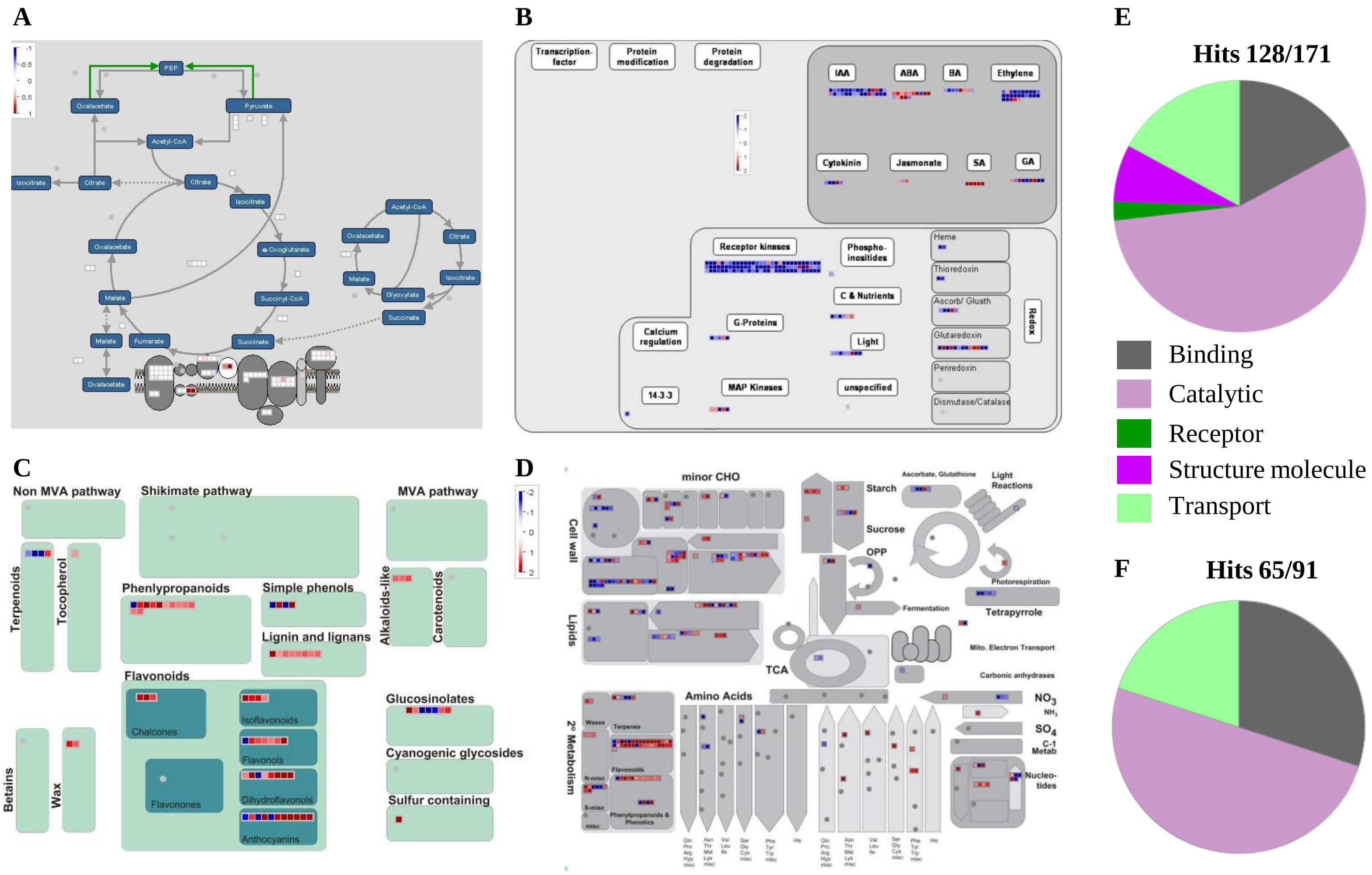

Figure-S4: Mapman and Go analysis for *pTSPO-PDC1-5* compare to WT plants grown under control conditions

(A-D) Mapman analysis summarizing the putative metabolic changes in transgenic plants compared to WT plants. **A**; Glycolysis, (**B**); Regulation overview, (**C**); Secondary metabolism, (**D**); Metabolic overview. Genes up-regulated in *pTSPO:PDC1* are shown as red, while the genes down-regulated are shown as blue.

(E-F); GO enrichment analysis. (**E**); Genes up-regulated, (**F**); down-regulated in *pTSPO:PDC1-5*. Go functional analysis for protein were performed using the panther classification system maintained at http://pantherdb.org/

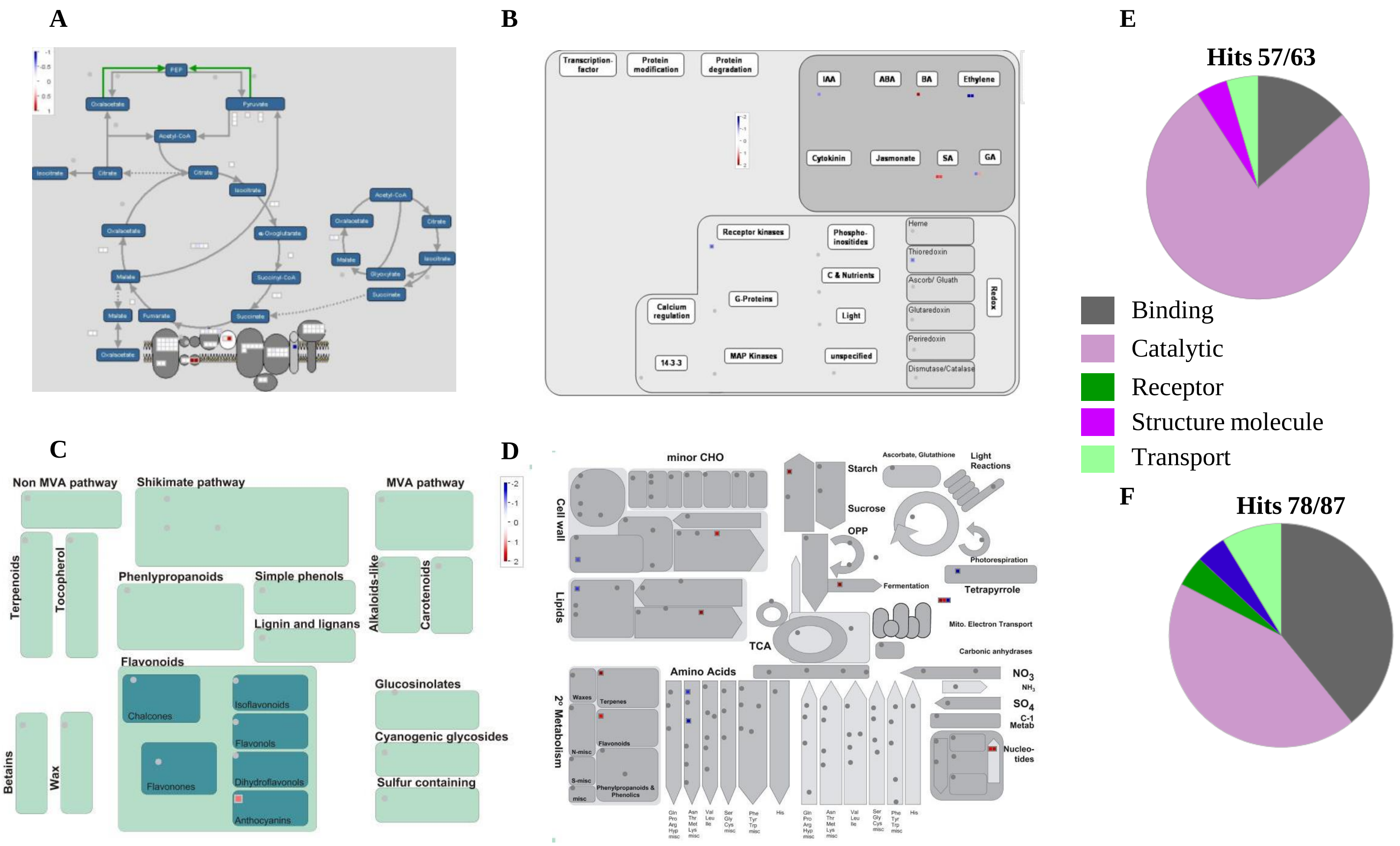

Figure-S5: Mapman and Go analysis for *pTSPO-PDC1-5* compare to WT plants grown under drought stress conditions

(A-D): Mapman analysis summarizing the putative metabolic changes in transgenic plants compared to WT plants. **A**; Glycolysis, (**B**); Regulation overview, (**C**); Secondary metabolism, (**D**): Metabolic overview. Genes up-regulated in *pTSPO:PDC1* are shown as red, while the genes down-regulated are shown as blue.

E-F; GO enrichment analysis. (**E**); Genes up-regulated, (**F**); down-regulated in *pTSPO:PDC1-5*. Go functional analysis for protein were performed using the panther classification system maintained at http://pantherdb.org/

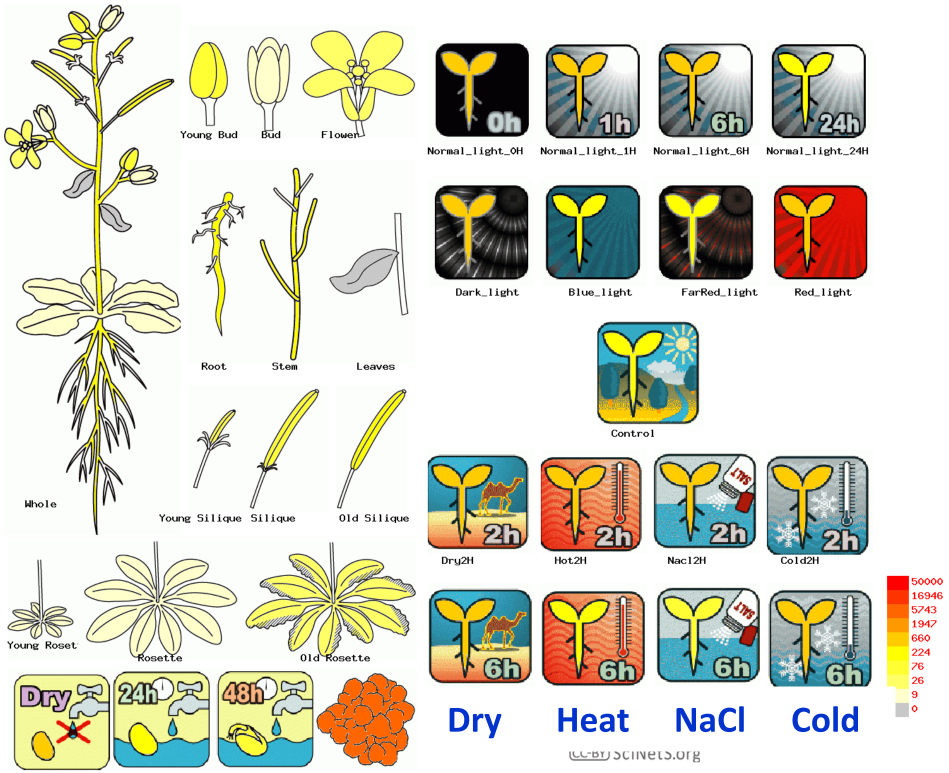

(CC-BY) SciNetS.org

Figure-S6: Expression profile of *PDC1* in Arabidopsis

The data was acquired from HanaDB-AT maintained at http://evolver.psc.riken.jp/seiken/search.html

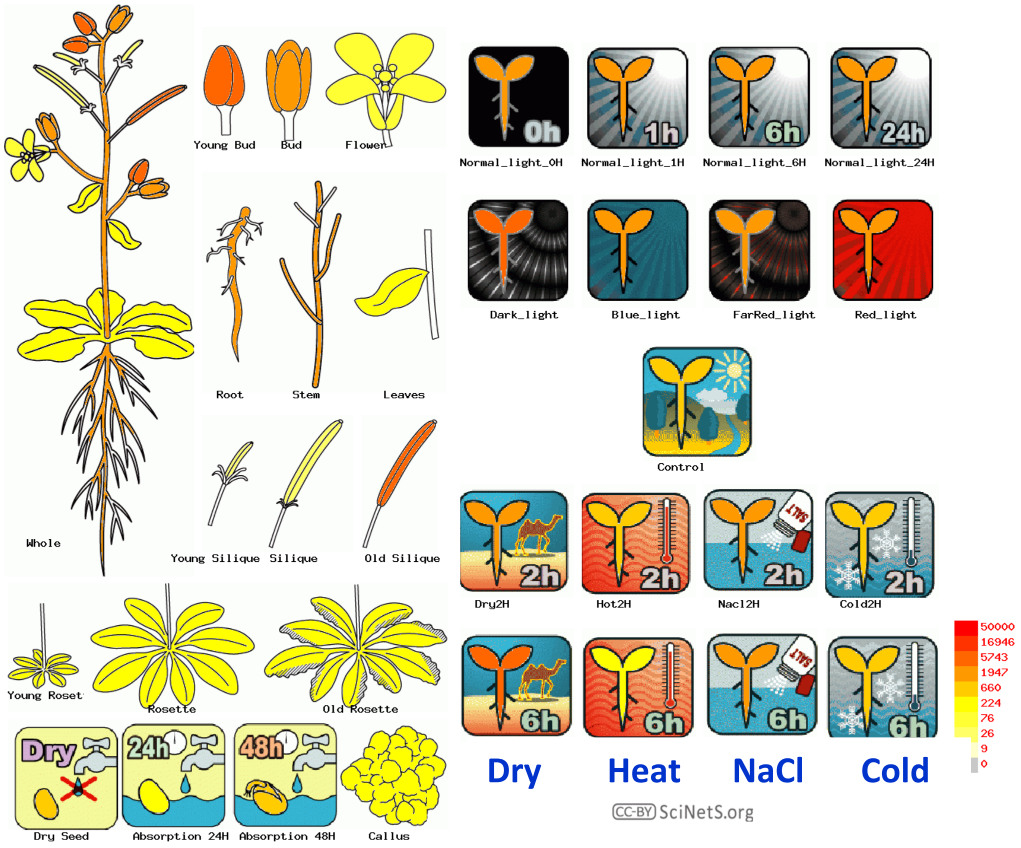

Figure-S7: Expression profile of *ALDH2B7* in Arabidopsis

The data was acquired from HanaDB-AT maintained at http://evolver.psc.riken.jp/seiken/search.html

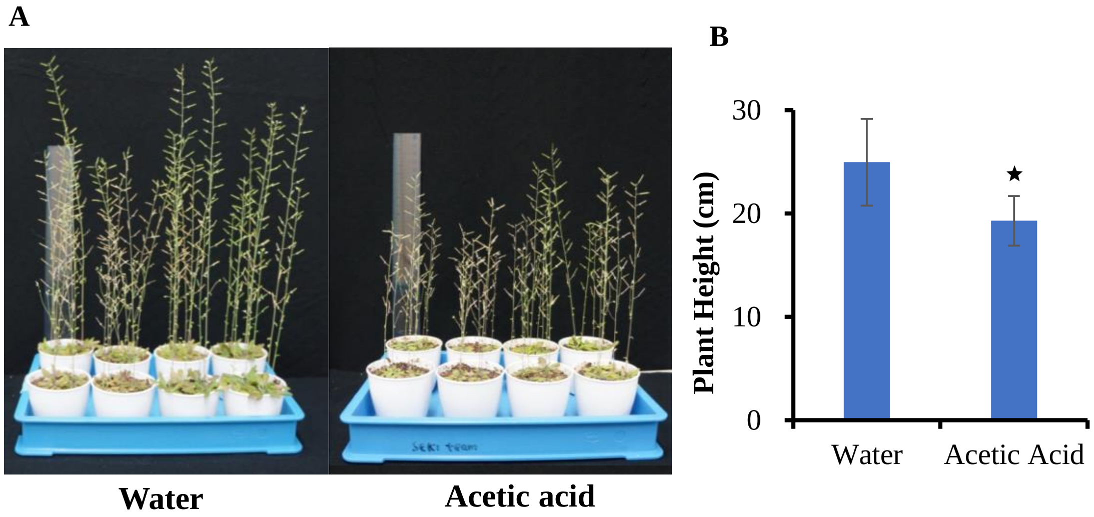

Figure-S8: Continuous Supply of Acetic acid hinders crop growth
15 days old plants were treated with water, or 10mM acetic acid for five weeks. Treatment was repeated once a week.
A: 8 week old plants, **B**: plant height (cm).
The column bar followed by asterisk is significantly different from WT plants ($p<0.05$). n=20.
